# Genomic Insights Into the Evolution of Parental Care in Weevils

**DOI:** 10.1093/gbe/evag142

**Published:** 2026-06-12

**Authors:** Sarah Rinke, Peter Biedermann, Martin Schebeck, Mark C Harrison

**Affiliations:** Institute for Evolution and Biodiversity, University of Münster, Münster, Germany; Chair for Forest Entomology and Protection, University of Freiburg, Stegen, Germany; Department of Forest Entomology, Faculty of Forest Sciences and Forest Ecology, University of Göttingen, Göttingen, Germany; Institute of Forest Entomology, Forest Pathology and Forest Protection, Department of Ecosystem Management, Climate and Biodiversity, BOKU University, Vienna, Austria; Centre for Discoveries in Life Sciences, Coventry University, Coventry, UK

**Keywords:** weevils, parental care, relaxed selection, transcriptional regulation, sheltering

## Abstract

Parental care, a key step in the evolution of sociality, has evolved multiple times in insects, yet the molecular mechanisms underlying its emergence remain poorly understood. Weevils (Curculionidae) exhibit diverse parental care behaviors, from nest building to egg and larval attendance, making them an ideal system to investigate genomic changes associated with social behavior. We analyzed 13 high-quality weevil genomes, encompassing independent origins of egg and larval attendance, to test two predictions: (i) the sheltering hypothesis, where parental care relaxes selection on traits critical for independent larval survival and (2) the regulatory hypothesis, where behavioral shifts are driven by changes in transcriptional regulation. In support of hypothesis 1, we identified over 400 genes with evidence of significantly relaxed selection on the branches where egg and larval attendance evolved. In further support, we uncovered a significant number of convergent gene losses that coincided with both origins of larval attendance, particularly in genes linked to transcriptional regulation, metabolism and development. In contrast, positive selection and intensified selection were rare but contained multiple genes regulating gene expression, consistent with hypothesis 2. Together, these results suggest that parental care in weevils drives both simplification of larval traits through relaxed selection and convergent gene loss, and innovation in caregiving behaviors via adaptive changes in gene regulation.

Significance Parental care is a pivotal evolutionary innovation, yet its genetic basis in insects remains underexplored. By comparing genomes from weevil species with and without care behaviors, we reveal two key processes shaping the emergence of subsociality: relaxation of selection on genes linked to larval independence and adaptive evolution in genes regulating gene expression. This combination likely reflects reduced demands on protected larvae alongside fine-tuning of parental behaviors. Our findings highlight how simple social systems can evolve through both loss and innovation, offering a comparative framework for understanding social evolution across insects.

## Introduction

Parental care is a social phenotype that is widespread in many vertebrate lineages, especially within mammals and birds. Taking care of offspring increases the offspring’s chances of survival and reproduction while often reducing parental fecundity (Royle et al. 2012). The extent to which a species performs parental care depends strongly on the environment and the type and availability of food (Wilson 1975; Tallamy 1984; Tallamy and Wood 1986). Parental care in insects, where it has evolved in at least 13 orders (Eickwort 1981, as cited in Tallamy 1984), predominantly exists in species feeding on leaves which are exposed to predators and parasites, in species feeding on or living in wood to inoculate the wood with symbionts and transfer fungal spores to their offspring, as well as in insects feeding on dung, which is an ephemeral and highly competitive resource (Royle et al. 2012). In beetles, most families in which parental care has evolved feed on these food types (Biedermann and Nuotclà 2020), for instance, leaf beetles (Chrysomelidae), dung beetles (Scarabaeidae), and wood-boring weevils (within Curculionidae).

Insects display a broad range of social phenotypes from parental care, also called subsociality, up to the most complex social phenotype, eusociality, which evolved in three insect orders: ants, bees, and wasps within Hymenoptera, termites within Blattodea and atleast one species of ambrosia beetle (*Austroplatypus incompertus*) within Coleoptera. Eusociality encompasses the presence of alloparental care, overlapping generations and division of reproductive labor into reproductive and sterile castes (Wilson 1971). Studies of less socially complex lineages, such as subsocial species without castes or social species with monomorphic, flexible castes, are important for understanding the evolution of eusociality (Linksvayer 2010; Shuker and Simmons 2014; Kronauer and Libbrecht 2018). However, research on the molecular evolution of sociality in insects has traditionally focused on eusocial species, especially within Hymenoptera (eg Simola et al. 2013; Kapheim et al. 2015; Shell et al. 2021; Jones et al. 2023) and less frequently in Blattodea (Harrison et al. 2018; Ewart et al. 2024).

It has been proposed that the evolution of social traits in insects may be driven by different molecular mechanisms depending on the level of social complexity (Rehan and Toth 2015). These predictions include, for example, changes in gene expression patterns that affect behavioral shifts in simple societies, while greater adaptational changes in gene repertoire are expected to be involved in the evolution of divergent caste phenotypes in eusocial species (Linksvayer and Wade 2005; Rehan and Toth 2015). Most of these expectations of gene family size evolution, adaptive protein evolution and evolutionary changes in transcriptional regulation have been confirmed with comparative genomic and transcriptomic analyses as being important for the evolution of increasing social complexity within eusocial taxa, such as bees, ants, wasps (Hymenoptera) and termites (Blattodea) (Simola et al. 2013; Kapheim et al. 2015; Harrison et al. 2018; Shell et al. 2021). However, subsocial species are mostly missing in these studies so that mechanisms involved in the evolution of simple social behavior such as parental care could not be distinguished from those associated with the evolution of more complex social phenotypes (reviewed in Mikhailova et al. 2024).

There are two main hypotheses regarding the molecular mechanisms related to the evolution of parental care: Parental care itself is suggested to lead to less intense selection due to sheltering of the offspring. In this scenario, parents compensate for nonoptimized traits of their offspring, thus increasing chances of survival and decreasing selective pressure on the involved traits], eg immunity or growth rate. This can lead to the accumulation of slightly deleterious gene variants and a dependency on parental care as observed in *Nicrophorus vespilloides* (Mashoodh et al. 2023; Pascoal et al. 2023). A second hypothesis proposes that adaptive changes in gene expression can evolve relatively easily through modifications in the regulation of transcription and translation, often resulting in altered timing or tissue-specific gene expression (Romero et al. 2012). This shift in gene expression is often regarded as an early step in the evolution of sociality and may play a role in the transition from solitary lifestyle to parental care behavior (Rehan and Toth 2015).

Here, we aim to test which evolutionary mechanisms occur at the emergence of subsociality in beetles. More specifically, we investigate if there is evidence for genomic changes in transcriptional regulation mechanisms and a relaxation of selection, which are expected to occur at the emergence of subsociality. Additionally, we investigate evidence for adaptive evolution, which is proposed to play a role in the evolution of higher social complexity.

Although parental care evolved in numerous beetle families, the highest number of evolutionary origins of parental care exist in the “true weevils” (Curculionidae, six origins) (Biedermann and Nuotclà 2020). Weevils feed on a great variety of plant parts and fungal tissues and are often categorized based on larval feeding modes (Kirkendall et al. 2015). Sociality level often, but not always, correlates with feeding pattern. Generally, free living weevils have a less complex social lifestyle and feed on a variety of plant parts, like seeds (spermatophagy) and fleshy (not woody) plant tissues (herbiphagy), with some bark beetles feeding on phloem. Ambrosia beetles (also present in other beetle groups), on the other hand, have a more complex social lifestyle and live in woody tissues where they cultivate fungi (xylomycetophagy) (Kirkendall et al. 2015). However, *Hypothenemus hampei* larvae, for example, feed on seeds, while their parental care behavior is more complex than in other species with a similar feeding mode. Complex social systems, like (facultative) eusociality, evolved in bark and ambrosia beetles, which is generally not the case in most free-living weevils (Biedermann and Nuotclà 2020).

Parental care behavior in weevils is less well explored than, eg in carrion beetles, as most species with parental care have a cryptic lifestyle, although more detailed observations are becoming available (Biedermann and Nuotclà 2020). Nevertheless, a simple classification into no care, nest building, egg attendance, and larval attendance is possible for most weevil species with published genomes (Kirkendall et al. 2015). Nest building by itself is the simplest form of parental care and can be defined by concealing the eggs from their surroundings by placing them in or under plant materials to prevent predation (Royle et al. 2012). Free living weevils do not build complex nests, but they lay their eggs in well-protected environments for their offspring and often cover the eggs, eg by rolling leaves around them. Nest building is relatively simple in species like the *Ips* bark beetles that mate in the phloem and lay individual eggs that they cover with boring dust, but more advanced in the fungus farming ambrosia beetles that live in groups (Kirkendall et al. 2015). In some species, parents stay with their eggs at the oviposition site until the larvae hatch, to protect and groom the eggs. This behavior is called prehatching care or egg attendance. Fewer species perform larval attendance, or posthatching care, where parents attend to their hatched larvae to protect and groom them (Royle et al. 2012).

**Fig. 1.**
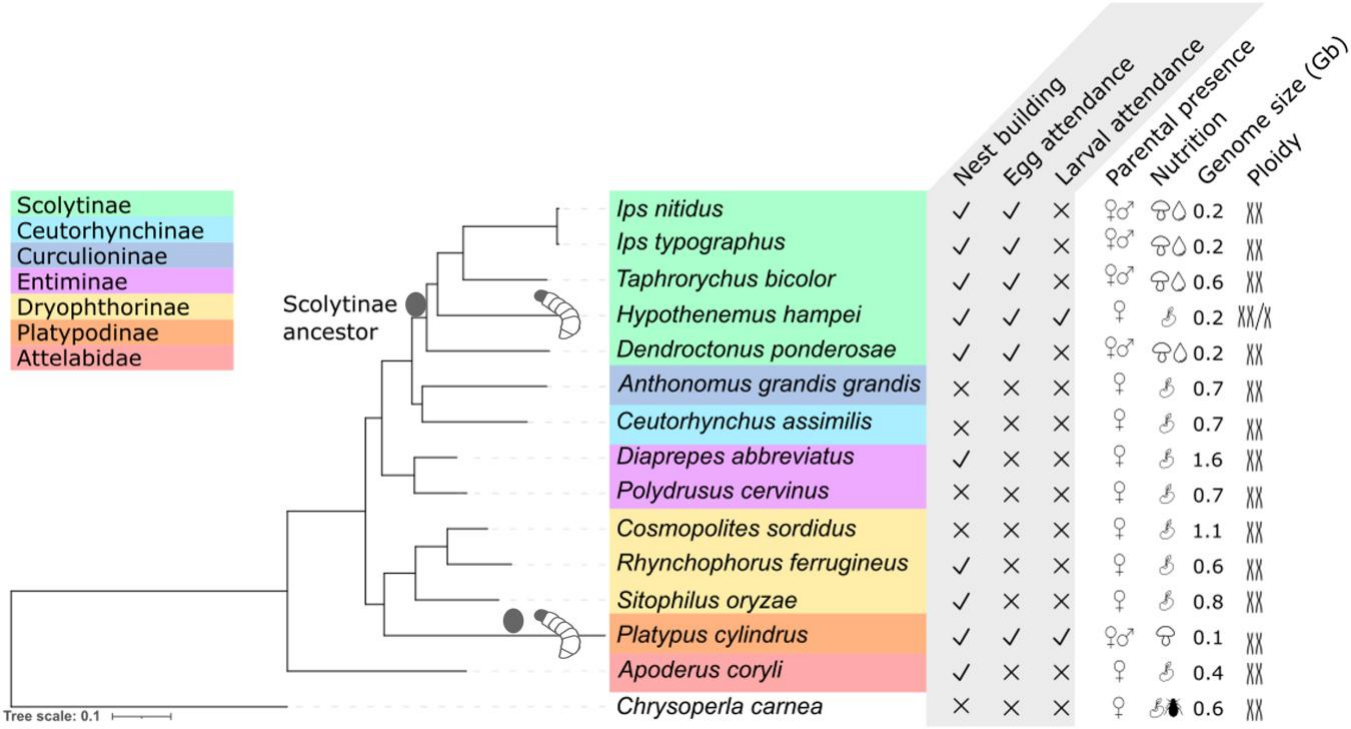
Phylogenetic tree of the studied species with information on parental care, nutritional, and genomic traits. The colors indicate the weevil subfamily, eggs indicate the evolution of egg attendance, while the larvae indicate the evolution of larval attendance. The ancestral branch at which egg attendance evolved within Scolytinae is indicated as “Scolytinae ancestor.” Form of care: all beetles in this tree select the oviposition site (not shown in figure). Nest building, egg attendance, and larval attendance are shown as present (✓) or absent (x). Parental presence is either uniparental female or biparental. The different nutrition types are indicated as follows: fungus and phloem, plant material, fungus, plant material, and aphids. Genome sizes are shown in gigabases. Ploidy is indicated as either diploid or haplodiploid.

Bark and ambrosia beetle species cover the sociality spectrum from gregariousness, via parental care, all the way to eusocial societies (Costa 2006). Furthermore, due to their pest behavior, a large number of published weevil genomes are publicly available, allowing evolutionary analyses into the emergence of social phenotypes in this group. In this study, we carried out evolutionary analyses on 13 weevil species with high-quality published genomes, covering the parental care phenotypes nest building, egg attendance, and larval attendance (Fig. 1). Larval attendance, which represents the most complex social phenotype in this study, has emerged twice within the species we included, allowing us to investigate convergent evolutionary mechanisms involved in the evolution of parental care. We employed selection and gene family size evolution analyses to test two hypotheses on the evolution of parental care in weevils: (i) that parental care relaxes selection on traits critical to survival in independent larvae and (ii) that behavioral shifts in parents are driven by adaptive changes in transcriptional regulation.

## Results

### Dataset

Only high-quality genomes with a BUSCO (Manni et al. 2021) completeness score ≥97 were included in our analyses. All genomes were reannotated to ensure uniform proteome quality for downstream analyses. The reannotated proteomes showed high levels of completeness >95 (Supplementary Table S1), measured with BUSCO and DOGMA (Dohmen et al. 2016). After quality filtering, our dataset included genomes of 13 species from the family of true weevils (Curculionidae), along with two outgroups, *Apoderus coryli*, belonging to the leaf-rolling weevils (Attelabidae) and one noncoleopteran species, the green lacewing *Chrysoperla carnea* (Fig. 1).

The analyzed weevils span a variety of levels of three parental care traits: nest building, egg attendance, and larval attendance. Nest building is a highly variable trait, ranging from depositing eggs in a protected environment and potentially covering them, to building elaborate galleries. Due to high variability in occurrence within our dataset, we were unable to reliably infer independent evolutionary origins of nest building with ancestral reconstruction (Fig. S1(a and b)). We therefore focus our evolutionary analyses on the origins of egg and larval attendance. Within our data set, egg attendance, the grooming and protection of eggs, is only performed by members of the Scolytinae subfamily and *Platypus cylindrus* (Platypodinae). Ancestral reconstruction indicates that egg attendance likely evolved in *P. cylindrus* and the ancestor of all Scolytinae species in this dataset (Fig. S1(c)). Within our dataset, adults of only two species, *H. hampei* and *P. cylindrus*, attend to their offspring after hatching.

Thus, the focal branches in this study are those on which egg attendance and larval attendance evolved (marked with eggs/larvae in Fig. 1, supported by ancestral reconstruction Fig. S1(d)). In most species, only mothers are present at the oviposition site or nest (maternal presence), with biparental presence occurring in five species (Fig. 1). The food sources range from different plant tissues in most species to fungus farming in ambrosia beetles. Genome sizes vary between 0.1 gigabases in *P. cylindrus* and 1.6 gigabases in *Diaprepes abbreviatus*. All species are diploid, except for *H. hampei*, which is haplodiploid (Fig. 1).

### Gene Family Size Evolution

**Fig. 2.**
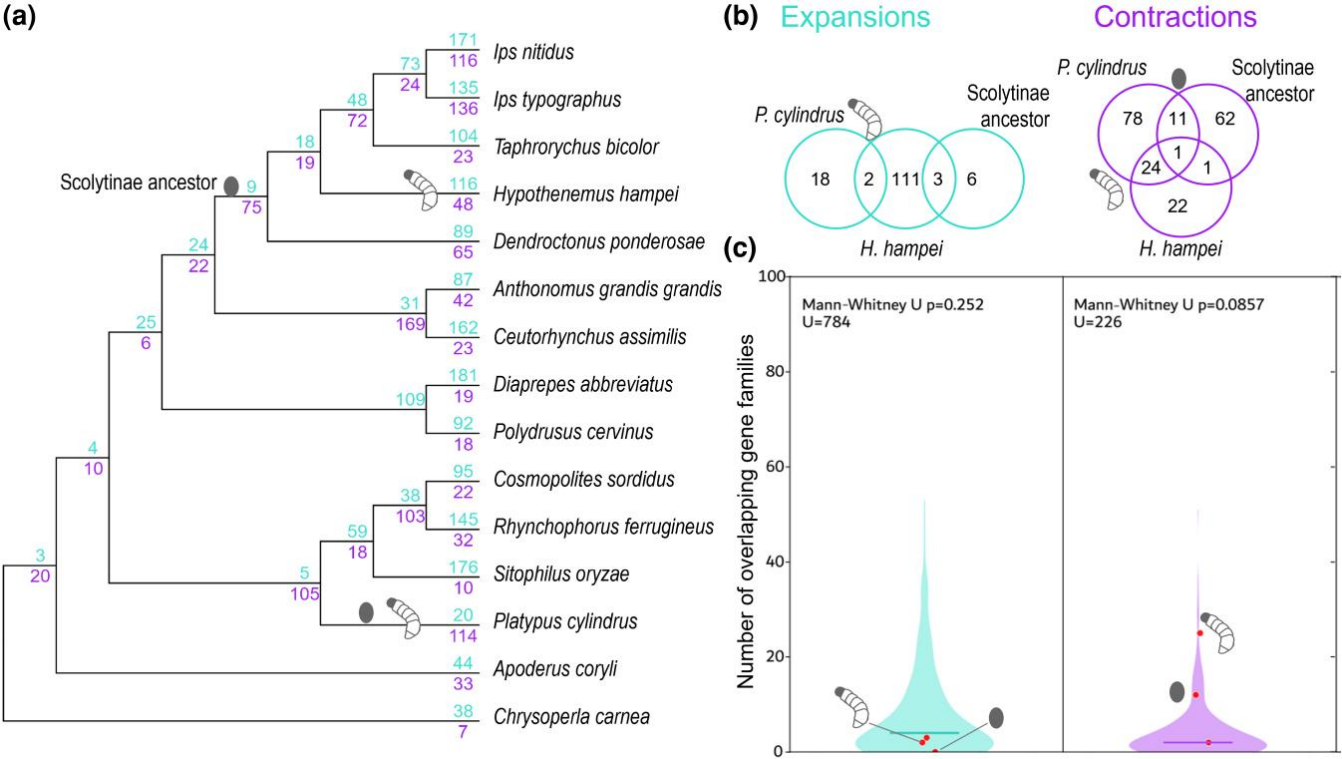
Gene family size changes across the phylogenetic tree and convergent contractions and expansions on branches of interest. CAFE5 was used to calculate expansions and contractions per branch. a) Phylogenetic tree showing all species in the dataset. All turquoise numbers (above branches) indicate gene family size expansions, while purple numbers (below branches) show contractions. Eggs denote where egg attendance evolved, larvae denote where larval attendance evolved. b) Venn diagrams of convergent expansions (left, turquoise) and contractions (right, purple) on the three branches where egg and/or larval attendance evolved. Egg/larva next to overlap indicate the parental care trait which is inferred to have evolved on both branches. c) Distribution of number of gene families convergently expanded or contracted (left—expansions, right—contractions) in all possible combinations of two branches across the tree. Median is indicated as a horizontal bar. Red dots indicate the number of observed convergent gene family size changes on two branches of interest (Scolytinae ancestor, *Hypothenemus hampei*, *Platypus cylindrus*). The egg/larva icon indicates which care trait is inferred to have evolved on the two branches of interest and correspond to the indicated overlaps in (b).

We analyzed gene family size changes across the phylogenetic tree to see if expansions and contractions of gene families correlate with differences in egg and larval attendance. For this, we used OrthoFinder (Emms and Kelly 2019) to cluster protein sequences into orthologous groups (∼ gene families). CAFE5 (Mendes et al. 2021) was then used to reconstruct ancestral sizes of gene families throughout the phylogeny in order to infer changes in gene family size. We focus on three branches of interest (marked with egg and larva symbols in Fig. 2): (i) the branch leading to the Scolytinae ancestor, on which we infer egg attendance to have evolved, (ii) the terminal branch of *H. hampei*, along which larval attendance is inferred to have evolved, and (iii) the terminal branch of *P. cylindrus*, on which both egg and larval attendance are inferred to have evolved. On the branch leading up to all Scolytinae species, 9 gene families were expanded, while 75 were contracted. In *H. hampei*, there were 116 gene family expansions and 48 contractions, while in *P. cylindrus*, 20 gene families were expanded and 114 were contracted (Fig. 2a). Genome assembly quality, both in terms of contiguity (N50) and assembly level (chromosome, scaffold, contig), had no significant effect on numbers of inferred expansions and contractions (Figs. S2 and S3).

To infer convergent evolution of gene families along with the emergence of egg and larval attendance, we considered numbers of gene families significantly expanded or contracted on more than one of the three branches of interest (Fig. 2b). Of particular interest for inferring convergent family changes were the pairs of branches which shared trait origins, ie egg attendance (*P. cylindrus* + Scolytinae ancestor) and larval attendance (*H. hampei* + *P. cylindrus*). We compared these values to expectations based on shared expanded and contracted gene families among all combinations of two branches across the tree (Fig. 2c). Overall, the number of convergent expansions (shared gene families) between two branches where egg and/or larval attendance evolved (*H. hampei* and Scolytinae ancestor: 3, *H. hampei* and *P. cylindrus*: 2, *P. cylindrus* and Scolytinae ancestor: 0) was lower than expected based on the numbers of convergent expansions found in all combinations of two branches (median: 4; Fig. 2b, c). Convergent contractions, on the other hand, were substantially higher than expected (median: 2 among all pairs of branches, Fig. 2c) between *P. cylindrus* and the Scolytinae ancestral branch (12) and between *P. cylindrus* and *H. hampei* (25), where egg and larval attendance convergently evolved, respectively (Fig. 2b). In contrast, only two gene family contractions were shared by the Scolytinae ancestral branch and *H. hampei* which do not share novel traits. These results indicate that convergent gene losses, but not gains, coincided with each of the two evolutionary origins of egg and larval attendance.

**Fig. 3.**
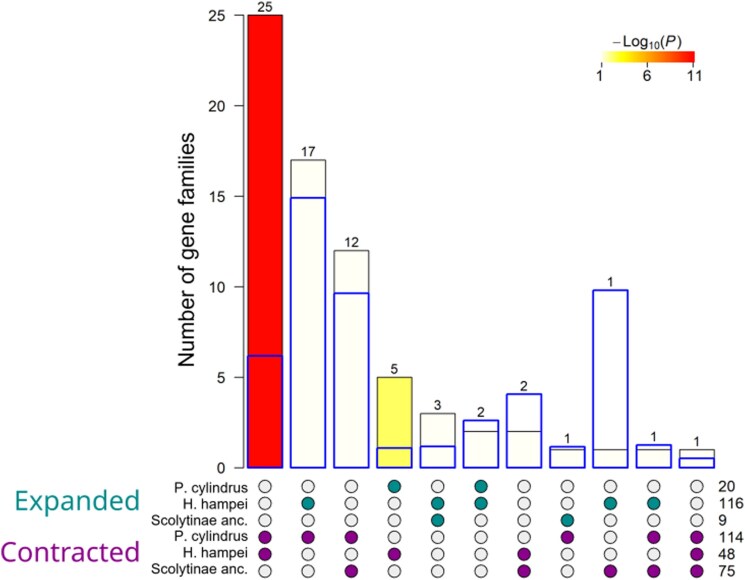
Superexact test of overlaps of gene family expansions and contractions on the three branches of interest, namely Scolytinae ancestor, *Platypus cylindrus* and *Hypothenemus hampei*. Filled circles at the bottom of the figure indicate, which expansions (turquoise, top 3 rows) and contractions (purple, lower 3 rows) are compared. The total number of expansions/contractions for each branch of interest is shown in the bottom right. Blue boxes indicate the expected overlap calculated from the superexact test. Yellow and red shading indicate the *P*-value is significant at P≤0.05 level.

A superexact test showed the convergent contraction of 25 gene families (including the one gene family convergently contracted on all three branches of interest) on the *P. cylindrus* and *H. hampei* branches, where larval attendance evolved, to be significant (expected: 6.17; $P=1.18\times 10^{-11}$; Fig. 3) compared to all other possible overlaps between two or more branches. Functions of these convergently contracted gene families could be related to the evolution of larval attendance, which emerged on each branch. We were able to infer putative functions for 20 of these 25 gene families revealing abroad range of functions (Table S2) including, among others, gene families involved in transcriptional regulation (zinc finger protein, histone 2A, endonuclease), metabolism (eg lipases, choline dehydrogenase, fatty acyl-CoA reductase, peptidases), and development (craniofacial development proteins). No GO-terms were enriched within these gene families underlining the diverse functions of these convergently contracted genes.

The gene family that was convergently contracted on all three branches of interest, and therefore could be related to the emergence of egg and larval attendance, is a lipase. One of the three gene families that are convergently expanded on *P. cylindrus* and *H. hampei* is Cytochrome P450, which plays an important role in detoxification in weevils (Blomquist et al. 2021). However, Cytochrome P450s are significantly expanded and contracted on multiple branches in our dataset, seemingly independent of care traits and possibly linked to varied functions and species-specific traits (Fig. S4).

### Selection

To understand whether the emergence of subsociality has an effect on selection strength, we analyzed dN/dS on 2,056 single copy orthologs across the phylogenetic tree using codeml from the paml software package (Yang 2007).

We observed strong purifying selection (median dN/dS <0.05) across the entire phylogenetic tree, with no visible differences on the branches where egg or larval attendance evolved (*H. hampei*—median: 0.03; *P. cylindrus*—median: 0.04; Scolytinae ancestor—median: 0.04), compared to all other weevil branches (medians: 0.00–0.04; Fig. S5). This indicates that there is no pervasive relaxation of selection across all tested orthologues on the branches where egg attendance and larval attendance evolved.

#### Relaxation and Intensification of Selection

To test if there are specific genes under relaxed or intensified selection on the three branches of interest compared to all other branches, we used RELAX (Wertheim et al. 2015) to analyze whether a change in strength of selection occurred with the emergence of parental care. Overall, we found a strong signal of relaxed selection on the branches where egg and larval attendance evolved (Fig. S6), with 436 genes under significant relaxed selection and only 38 under intensified selection. The functional annotation of the top 10 genes under relaxed selection on the three branches where egg and larval attendance evolved (Table S3), revealed two clusters of functions: (i) gene expression regulation, more precisely—translation elongation, regulation of transcription, RNA processing (HELICc2), and pre-rRNA processing (rRNA adenine N(6)-methyltransferase family) and (ii) neural plasticity, namely dystrobrevin binding protein 1 and KIF-1 binding protein. Two other proteins could be annotated with functions related to protein binding and signal transducer activity, while two more proteins have unknown functions (C-terminal to LisH motif and unknown). Three of the top 10 genes under intensified selection (Table S3) are related to gene expression regulation—specifically pre-mRNA splicing (LSM4 homolog), regulation of transcription and regulation of mRNA processing. Another protein, with the broad function “Cytoskeleton/chromatin conformation” might also be involved in transcriptional regulation through the changes in chromatin conformation. Two proteins are involved in protein transport, namely ADP-ribosylation factor family and peptidase S24-like. Another two proteins are involved in electron transfer: namely ATP-dependent (S)-NAD(P)H-hydrate dehydratase and the mitochondrial ubiquinol-cytochrome C chaperone. Two further proteins play a role in carbohydrate metabolism: chitin metabolism and N-acetylgalactosaminyltransferase.

To test if parental care correlates with intensification or relaxation of selection across the phylogenetic tree, we ran RELAX for each single copy orthogroup with each branch set as the foreground branch. The proportion of orthogroups under significant relaxed selection was much higher in *P. cylindrus* (18.73%) than on all other branches, while *H. hampei* had the fourth highest value (4.86%, Fig. S8(a)). On the other hand, the proportion of genes under intensified selection was very low in *P. cylindrus* (0.44%) and *H. hampei* (0.97%), the second and fourth lowest value, respectively (Fig. S8(b)). Genes under relaxed selection on these focal branches are functionally enriched for the following GO terms: regulation of gene expression, regulation of cellular biosynthetic process, regulation of biological process, protein modification process, regulation of transcription by RNA pol II, cellular process, and DNA-templated transcription.

**Fig. 4.**
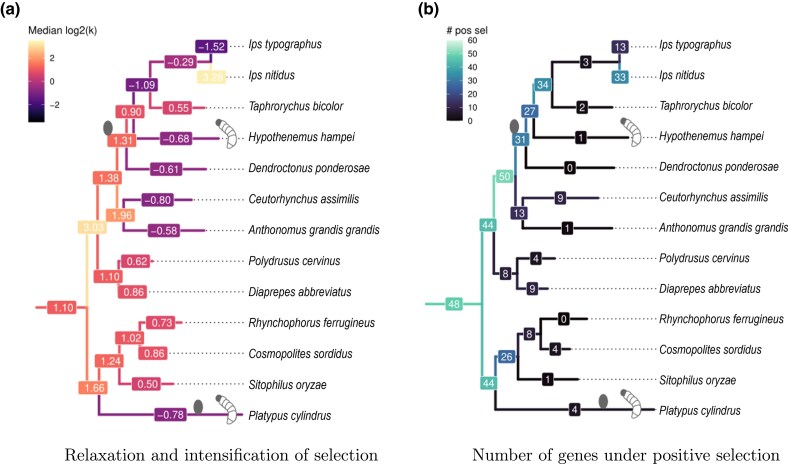
a) Phylogenetic tree showing median log2(k) values for each branch. Eggs indicate where egg attendance evolved, while larvae indicate where larval attendance evolved. Positive values indicate intensification, negative values relaxation of selection. log2(k) was calculated for all single copy orthologues for each branch separately. For each branch, the median value of all significant (p.adjust) *k* values was then calculated. b) Phylogenetic tree showing the number of genes under positive selection on each branch. a) Relaxation and intensification of selection and b) number of genes under positive selection.

These results are reflected in the median relaxation–intensification coefficient, log2(k) (Fig. 4(a)). A positive log2(k) indicates intensification of selection, while a negative value indicates relaxation of selection. Both terminal branches with larval attendance have negative log2(k) values (*H. hampei*: −0.68, *P. cylindrus*: −0.78), but there are also species without parental care with a negative log2(k). We formally tested if there is a correlation between parental care and strength of selection, which takes phylogenetic signals in the data into account. For this, we performed a phylogenetic generalized least squares analysis (PGLS) for median *k* per branch (Fig. 4(a)), comparing two models: mediank∼parentalcare,correlation=corBrownian(1,  tree,form=∼species) and mediank∼parental  care,  correlation=corMartins(1,tree,form=∼species). The second model (OU model) had a better fit according to log Likelihood score (20.51710) and AIC (−29.03419) (Table S5). According to this model, larval attendance has a significantly lower median intensification and relaxation parameter than nest building after multiple testing correction (Tables S6, S7, Fig. S7). This difference indicates that branches, on which larval attendance evolved, experienced a relaxation of selection compared to branches with nest building as the only parental care phenotype. However, we find no evidence for a relaxation in selection related to egg attendance.

#### Positive Selection

To test if positive selection plays a role in the evolution of egg or larval attendance, we used aBSREL (Smith et al. 2015) to calculate for each branch, how many genes showed signs of significant positive selection. On the branches where egg and larval attendance evolved, there were not more genes under positive selection than on other branches, namely 31 on the branch leading up to all Scolytinae, 4 on *P. cylindrus* and 1 on *H. hampei*, compared to 0–33 genes on all other terminal weevil branches (3–50 genes on internal branches, see Fig. 4(b)). Furthermore, no genes were under convergent positive selection on more than one of the branches of interest. However, functional annotation (see Table S4) revealed multiple genes related to transcriptional regulation to be under positive selection in *P. cylindrus* (three out of four genes) and the ancestor of Scolytinae (6 out of 31 genes), hinting at a possible role of changes in gene expression regulation in the evolution of egg attendance. Additionally, positive selection was observed in genes connected to microtubules and cytoskeletal structures in *H. hampei* and the Scolytinae ancestor. Interestingly, Moesin ezrin radixin and ATP-dependent microtubule severing protein, under positive selection in *H. hampei* and the Scolytinae ancestor, respectively, both seem to be involved in axon targeting and axon regeneration. Together with the dystrobrevin binding protein 1 associated with neural plasticity under relaxed selection on all three branches of interest (Table S3) these findings indicate that neural development may be involved in the evolution of parental care. Genes under positive selection in the ancestor of all Scolytinae can be grouped into further functional categories, such as DNA and protein-related processes, calcium homeostasis, microtubule related processes, respiratory electron chain, and ion binding (Table S3).

## Discussion

Subsociality, in which adults provide care for their offspring, is often regarded as a prerequisite for more socially complex phenotypes, such as eusociality, to evolve (Rehan and Toth 2015). The evolution of subsociality in insects is expected to coincide with changes in transcriptional regulation that influence caring behavior, while adaptive evolutionary processes are hypothesized to play a greater role in the evolution of more complex sociality (Rehan and Toth 2015; Pyenson and Rehan 2024). Furthermore, caring for dependent larvae may lead to a relaxation of selective forces on functions that are more important for survival in free-living, independent larvae (Mashoodh et al. 2023). In the true weevil family (Curculionidae) various subsocial traits have evolved multiple times, while eusociality exists in a single species, making this an ideal clade for studying the molecular evolution of simple social phenotypes. We analyzed genomes of 13 weevil species that span multiple combinations of care behaviors, including two independent origins of egg and larval attendance, to investigate the genomic mechanisms involved in the evolution of subsociality in weevils.

We performed analyses of selection and gene family size evolution to test two main hypotheses: (1) the sheltering effects of parental care lead to relaxed selection on genes critical for larval survival; (2) the emergence of caring behavior in weevils is associated with the adaptive evolution of transcriptional regulation.

### Sheltering of Larvae Leads to Relaxed Selection and Gene Losses in Species with Larval Attendance

We expected parental care to act as a selective buffer in *P. cylindrus* and *H. hampei*, reducing the need for offspring to evolve traits that are necessary for independent larvae to survive in unprotected conditions. In support, we find over 400 genes to have experienced a significant relaxation of selection strength on the branches where egg and larval attendance evolved, compared to only 38 genes with intensified selection. When comparing across the phylogeny, selection was significantly more relaxed in the two species with larval attendance, compared to species with just nest building, emphasizing the sheltering effect of parental care of larvae.

Mainly genes involved in transcriptional regulation and neural plasticity were under relaxed selection. The former may be associated with an overall reduction in protein synthesis linked to a reduced spectrum of behaviors in protected larvae. The latter may be related to changes in early brain development of dependent larvae as suggested for dependent larvae in burying beetles (Capodeanu-Nägler et al. 2016).

In further support of a link between relaxed selection and larval attendance, we found more convergently contracted gene families on branches where parental care evolved than on other branches. This was especially pronounced on the two branches where larval care evolved, indicating a loss of common functions related to parental care led to a convergent loss of genes from a high number of gene families. These 25 contracted gene families represented a broad range of functions, such as transcriptional regulation, metabolism, and development, which may again be related to an overall reduction in the functional diversity of larvae with dependence on parental care (Capodeanu-Nägler et al. 2016).

Overall, across all single copy orthologs, we found genome-wide uniform strong purifying selection across the phylogenetic tree, including the species with parental care. This indicates that relaxed selection in weevils with larval attendance only affects specific functions, possibly those linked to larval survival, rather than being pervasive across the genome as a consequence of reduced effective population size, as has previously been suggested for eusocial species (Weyna and Romiguier 2021; Ewart et al. 2024; Roux et al. 2024). While our analyses support that a significant relaxation of selection occurred with the evolution of larval care, we acknowledge that some of the involved genes may be attributable to other species-specific traits shared by *P. cylindrus* and *H. hampei*.

### Evidence for Adaptive Changes in Transcriptional Regulation Along with the Emergence of Egg and Larval Attendance

Adaptive evolution is hypothesized to play a greater role in the emergence of more advanced levels of insect sociality, than in the transitions from solitary to subsocial lifestyles, where mainly changes in transcriptional regulation are expected (Rehan and Toth 2015).

Accordingly, we find evidence of positive selection only in few genes (36) on the three branches where parental care of larvae or eggs evolved in our dataset. However, consistent with expectations for the evolution of subsociality, many of these genes under positive selection are involved in transcriptional regulation. Specifically, in *P. cylindrus*, where egg and larval attendance evolved, we found that three out of four genes under positive selection had functions related to transcriptional regulation, namely orthogroups with sequence-specific DNA binding function, zinc finger protein 512B and a gene containing an high mobility group (HMG) box, all of which are involved in DNA binding during transcription.

In the ancestor of all Scolytinae species in this study, in which egg attendance evolved, we found 31 genes under positive selection, covering a broader range of functions. Six of these genes have functions related to gene expression regulation and another eight genes have functions related to DNA and protein related processes. In *H. hampei*, where only larval attendance evolved, we found only one gene, Moesin ezrin radixin homolog, to be under positive selection. This gene plays a role in different processes, including mRNA export, axon targeting, and connections of cytoskeletal structures and the plasma membrane (McCartney and Fehon 1996; Ruan et al. 2013; Kristó et al. 2017).

We also found a relatively low number of genes under intensified selection along with the evolution of egg and larval attendance. The top 10 genes with the strongest signal of intensified selection included three genes involved in transcriptional regulation: LSM4 homolog, likely involved in pre-mRNA splicing and a gene containing a domain found in Pit-Oct-Unc transcription factors likely involved in regulation of transcription, as well as a further gene involved in the regulation of mRNA processing. Additionally, a gene belonging to the actin family might play a role in transcriptional regulation through chromatin conformation changes. This suggests that transcriptional regulation plays an important role in the evolution of parental care. Other genes under intensified selection had functions related to protein transport, electron transfer, and carbohydrate metabolism.

These results support the expectation of adaptive evolution playing a subordinate role in the evolution of parental care compared to changes in gene expression patterns. Furthermore, we present evidence for the adaptive evolution of multiple genes related to transcriptional regulation coinciding with the emergence of egg and larval attendance, which may be related to changes in parental behavior. However, despite these promising findings, we do not find adaptive signals for transcription genes at the origin of larval care in *H. hampei*, while at the origin of egg attendance in Scolytinae we detect a broader set of functions under positive selection besides transcriptional regulation. The development and analysis of transcriptomic data for multiple lineages with varying caring behaviors is necessary to further investigate adaptive changes in gene expression patterns.

### Conclusion

Our findings support the hypotheses that parental care contributes to sheltering, manifested as relaxed selection in a subset of genes, and that changes in parental care behaviors may drive adaptive evolution in transcriptional regulation. Specifically, we observed gene loss or reduced functionality in genes associated with transcriptional regulation, suggesting relaxed selection in larvae whose behavioral repertoires may be reduced. Conversely, we presented partial support for an adaptive evolution or intensification in other regulatory genes, which may underpin behavioral innovations linked to parental care. These findings partially overlap with those linked to advanced eusocial phenotypes (Mikhailova et al. 2024), underscoring the need for finer representations of social phenotypes in genomic analyses to better capture the molecular processes at each stage of social evolution.

We propose that these dual trajectories, relaxed selection and adaptive evolution, reflect a fundamental tension in the evolution of subsociality: simplification of dependent behaviors and elaboration of caregiving traits. Future comparative genomic and transcriptomic studies, especially across diverse lineages exhibiting parental care, will be crucial for disentangling these dynamics and separating general evolutionary trends from species-specific phenomena.

## Materials and Methods

In this study, we included published genomes of 13 weevil species covering different parental care phenotypes, as well as two outgroups (the green lacewing *Chrysoperla carnea* (Crowley 2021) and the leaf-rolling weevil *Apoderus coryli* (Crowley 2021)). The two species with the most elaborate parental care phenotype are *Platypus cylindrus* (Barclay et al. 2024) (Platypodinae) and *Hypothenemus hampei* (Navarro-Escalante et al. 2021) (Scolytinae) which independently evolved egg and larval attendance (Fig. 1). Four additional Scolytinae species with egg attendance, namely *Ips nitidus* (Wang et al. 2023), *Ips typographus* (Powell et al. 2021), *Taphrorychus bicolor* (Telfer and Badham 2024) and *Dendroctonus ponderosae* (Keeling et al. 2022) suggest that egg attendance evolved in the ancestor of the five Scolytinae species in this study. Three other weevil species from two weevil groups perform nest building, namely *Diaprepes abbreviatus* (Sylvester et al. 2024), *Rhynchophorus ferrugineus* (Dias et al. 2021), and *Sitophilus oryzae* (Parisot et al. 2021). All other species deposit their eggs without providing any further care: *Anthonomus grandis grandis* (Childers et al. 2021), *Ceutorhynchus assimilis* [Pest Genomics Initiative between Rothamsted Research, Bayer, and Syngenta], *Cosmopolites sordidus* (Rodriguez Ruiz and Van Dam 2023), and *Polydrusus cervinus* (Barclay et al. 2023).

### Data Acquisition

In January 2024, we downloaded all 24 available weevil genomes from the NCBI database (see Table S1). Of these genomes, 22 belonged to the true weevils (Curculionidae) and two belonged to the closely related outgroup of leaf-rolling weevils, *Apoderuscoryli* and *Cylas formicarius*. The genome of an additional outgroup species, *C. carnea*, a common green lacewing, was also obtained from NCBI. We checked assembly quality using BUSCO v 5.6.1 in genome mode with the insecta core set insecta_odb10 (Manni et al. 2021). Only genomes with a BUSCO completeness scores higher than 97% were reannotated for further analysis in this study.

### Reannotation

For the comparable annotation of all genomes, we followed the FastTE (Bell et al. 2022) pipeline for the repeat annotation and masking. Briefly, we used EDTA v2.1.3 (Ou et al. 2019) and DeepTE (Yan et al. 2020) for *de novo* TE annotation and classification, followed by repeat masking using RepeatMasker (Smit et al. 2015). The repeat-masked genomes were then trimmed using Trimmomatic (Bolger et al. 2014) to avoid adapter contamination. BRAKER 3.0.2 (Lomsadze et al. 2005; Stanke et al. 2006; Gotoh 2008; Stanke et al. 2008; Iwata and Gotoh 2012; Buchfink et al. 2015; Hoff et al. 2016, 2019; Brůna et al. 2020, 2021) was used for genome annotation using the OrthoDB v11 eukaryota protein set. The quality of the genome annotation was assessed using BUSCO (Manni et al. 2021) in protein mode and DOGMA (Dohmen et al. 2016). Using a DOGMA score of 90% and BUSCO score of 95% as quality thresholds, 15 species remained in this study (see bold names in Table S1).

Coding sequences (cds) were extracted using gffread from cufflinks 2.2.1 (Trapnell et al. 2010) and protein domains were annotated using PfamScan (Finn et al. 2014).

### Orthology, Alignment, and Phylogenetic Tree Construction

SequenceFairies seqCheck was used to remove stop codons from the reannotated proteome file and isoformCleaner to remove all but the longest isoform (Kemena 2024). Orthogroups were inferred using OrthoFinder v.2.5.4 (Emms and Kelly 2019) with default settings. Single copy orthologues were used for selection analyses, while multicopy orthologues were used for gene family size analyses. Proteome sequences were aligned with PRANK v.170427 (Löytynoja 2014). Subsequently, a phylogenetic tree was constructed using iqtree (Minh et al. 2020) and rooted with iTol v5 (Letunic and Bork 2021).

### Ancestral Reconstruction

Ancestral reconstruction for the parental care traits was performed using a maximum likelihood approach in a custom R script using the libraries phytools (Revell 2024), ape (Paradis and Schliep 2019), and geiger (Pennell et al. 2014).

### Gene Family Size Changes

A preliminary analysis showed that many of the expanded and contracted gene families had annotations that contained transposable elements (TEs). To ensure that these elements with large copy number variations do not interfere with the analysis of gene family size changes, we performed an additional filtering step on all proteomes. Specifically, we removed all genes containing the following Pfam domains: PF00075, PF00078, PF00665, PF02925, PF02992, PF03184, PF03221, PF03732, PF04687, PF05699, PF05840, PF05970, PF07727, PF08283, PF08284, PF10551, PF13358, PF13359, PF13456, PF13837, PF13976, PF14214, PF14223, and PF14529, taken from Min and Choi (2019). We also ran transposonPSI (Brian J. Haas, TransposonPSI, 2007–2011 <http://transposonpsi.sourceforge.net>) and combined both lists of genes. We removed these genes from the proteomes, and subsequently reran OrthoFinder as described above. As there were still some TE-related gene families in the dataset, we filtered out all orthogroups with domain annotations containing DDE, PiggyBac, YqaJ, reverse transcriptase, transposase, transposition, Phage integrase, transposable, and transposon. Gene family size changes across the phylogenetic tree were then analyzed using CAFE v5.0 (Mendes et al. 2021). The rooted phylogenetic tree was transformed to an ultrametric tree using the make_ultrametric.py script that comes with OrthoFinder. The clade_and_size_filter.py script was used to subset small and large gene families, subsequent analyses were performed on the small gene families. We ran CAFE v5.0. multiple times with different gamma values to see which gamma value has the highest final likelihood. In this case, gamma = 1 had the highest likelihood score. The testing of different lambda values did not make sense biologically, so we chose lambda = 1. The values obtained were then used for analysis of the large gene families, but the analysis failed due to too few large gene families. Results were plotted using CafePlotter (https://github.com/moshi4/CafePlotter). To test whether there is a correlation between N50 and significant gene family size changes, Pearson’s correlation coefficient was calculated using a custom R script. An ANOVA was used to test for an association between assembly level (contig, scaffold, chromosome) and significant expansions/contractions using a custom R script. A custom R script was used to analyze convergent contractions and expansions of gene families on three branches of interest. Functional annotation of convergently expanded and contracted gene families was performed using eggnog-mapper version 2.1.12 (Huerta-Cepas et al. 2019; Cantalapiedra et al. 2021) with eggNOG DB version: 5.0.2, diamond version 2.1.10 (Buchfink et al. 2021).

### Selection Analyses

For the selection analyses, the .codingseq files from the reannotation with BRAKER3 were used. Coding sequences for each single copy orthogroup were extracted using SequenceFairies seqExtract (Kemena 2024) and aligned based on the proteome files using pal2nal (v14) (Suyama et al. 2006). The coding sequence alignment was trimmed using Gblocks (0.91) (Castresana 2000) with the parameters -t = c -b5 = h. A custom python script was then used to format Gblock output files as in fasta format.

#### Estimation of dN/dS Values

The dN/dS values (ratio of the number of nonsynonymous substitutions per nonsynonymous site to number of synonymous substitutions per synonymous site), as an indicator for selective pressure, were calculated for each branch in the species tree and for each single copy orthologue. The calculation was performed using the free-ratios model implemented by CodeML in PAML suite (v. 4.9h) (Yang 2007). dN/dS values were extracted using a custom python script. Analyses were performed in R (R Core Team 2023) using RStudio (Posit team 2023). A custom R script was used to plot median dN/dS per branch on the phylogenetic tree using the packages ggtree (Yu et al. 2017, 2018; Yu 2020; Xu et al. 2022; Yu 2022), ggplot2 (Wickham 2016), viridis (Garnier et al. 2024), and treeio (Wang et al. 2020).

#### Test of Selection

To test if parental care is associated with a change in selection regime (relaxation or intensification of selection), RELAX (Wertheim et al. 2015) from the HyPhy suite (v. 2.5.6) (Pond et al. 2005) was used with default options. RELAX was run for each single copy orthogroup with the branches where parental care evolved, namely Scolytinae ancestor, *H. hampei* and *P. cylindrus*, set as foreground branches. Subsequently *P*-values were corrected for multiple testing using the false discovery rate (FDR). To compare the functions of the top 10 genes under relaxed/intensified selection on the branches of interest, genes were functionally annotated using eggnog-mapper version 2.1.12 with eggNOG DB version: 5.0.2, diamond version 2.1.10. The distribution of *k* (intensification and relaxation parameter) was plotted using a custom R script with the packages ggplot2 and dplyr (Wickham et al. 2023).

To generate data for linear modeling, we ran RELAX 27 times for each single copy orthogroup, each time with a different branch as foreground branch. A custom python script was used to extract genes under relaxed/intensified selection. The median-k-tree was plotted using a custom R script using the packages ggtree, ggplot2, viridis, dplyr and ape (Paradis and Schliep 2019). To test if the observed differences in median k are statistically significant, we used a phylogenetic generalized least square (PGLS) model with Brownian Correlation Structure as well as Martins and Hansen’s (1997) covariance structure (OU). The custom R script for these analyses made use of the libraries ape, nlme (Pinheiro and Bates 2000; Pinheiro et al. 2025), dplyr, emmeans (Lenth 2025), and treeio.

To test for positive selection of genes in the phylogeny, aBSREL (Smith et al. 2015) from the HyPhy suite (v.2.5.6) was used with default options. To test if the branches where parental care evolved are subject to episodic selection, we ran aBSREL with a priori selecting the foreground branches. Then, to perform an exploratory analysis, we ran aBSREL for each single copy orthogroup without foreground branches. A custom bash script was used to count the number of genes under positive selection per branch. Functional annotation was performed for the genes of interest as described above and a tree showing number of genes under positive selection was plotted using a custom R script using the same packages as for the median-k-tree.

## Supplementary Material

evag142_Supplementary_Data

## Data Availability

Genome assemblies were publicly available at NCBI (accession numbers see Table S1). Genome annotations, namely coding sequences (cds) and proteomes produced in this study are available at https://doi.org/10.5281/zenodo.16760856.
