# Supplementary material for: Genomic Insights Into the Evolution of Parental Care in Weevils
Source: Genome Biol Evol. 2026 Jun 12;18(6):evag142. doi: 10.1093/gbe/evag142 (PMC13289751; doi:10.1093/gbe/evag142)
Supplement: evag142_Supplementary_Data [file evag142_supplementary_data.pdf]

Genomic Insights into the Evolution of Parental Care in Weevils
Supplementary Materials

Sarah Rinke[1], Peter Biedermann[2], Martin Schebeck[3,4], and Mark C. Harrison[5,*]

[1]Institute for Evolution and Biodiversity, University of Münster, Münster, Germany
[2]Chair for Forest Entomology and Protection, University of Freiburg, Stegen, Germany
[3]Department of Forest Entomology, Faculty of Forest Sciences and Forest Ecology, University of Göttingen, Göttingen, Germany
[4]Institute of Forest Entomology, Forest Pathology and Forest Protection, Department of Ecosystem Management, Climate and Biodiversity, BOKU University, Vienna, Austria.
[5]Centre for Discoveries in Life Sciences, Coventry University, Coventry, UK
[*]Corresponding author: Mark.Harrison@coventry.ac.uk

Table S1: Quality assessment of genomes downloaded from NCBI and re-annotated proteomes using BUSCO and DOGMA. Species with BUSCO genome completeness score of >97%, BUSCO proteome completeness score of >95% and DOGMA completeness score of >90% were included in this study (bold). Analyses were not performed if previous steps failed or led to the exclusion of the species (-).

**Species**	**Accession Number**	**Genome size** (Gb)	**Genome quality** BUSCO (%)	**Proteome quality**		**Fragmentation**	
				BUSCO (%)	DOGMA (%)	N50 (MB)	Assembly level
Anthonomus grandis grandis	GCF_022605725.1	0.7	99.5	95.4	92.17	36.5	chromosome
Ceutorhynchus assimilis	GCA_917834065.1	0.7	98.7	95.6	92.71	55.4	chromosome
Cosmopolites sordidus	GCA_031761425.1	1.1	98.6	97.2	91.82	0.7	contig
Dendroctonus ponderosae	GCF_020466585.1	0.2	98.5	96.1	93.71	16.5	scaffold
Dendroctonus valens	GCA_024550625.1	0.3	96.2	92.6	89.65	-	-
Diaprepes abbreviatus	GCA_034092305.1	1.6	99.5	96.3	91.62	7.8	contig
Elaeidobius kamerunicus	GCA_014849505.1	0.3	55.1	-	-	-	-
Hypothenemus hampei	GCA_013372445.1	0.2	97.0	95.6	92.06	0.3	scaffold
Ips nitidus	GCA_018691245.2	0.2	97.4	95.6	92.20	16.4	chromosome
Ips typographus	GCA_016097725.1	0.2	99.2	97.7	94.74	6.6	contig
Kuschelorhynchus macadamiae	GCA_030620095.1	2.0	97.7	85.3	83.61	-	-
Listronotus bonariensis	GCA_014170235.1	1.1	93.3	71.6	73.51	-	-
Listronotus oregonensis	GCA_019359885.1	1.3	88.2	81.3	80.67	-	-
Orchestes rusci	GCA_958502075.1	0.6	99.6	94.5	91.13	-	-
Pachyrhynchus sulphureomaculatus	GCA_019049505.1	2.1	-	-	-	-	-
Pissodes strobi	GCA_016904865.1	2.0	-	-	-	-	-
Platypus cylindrus	GCA_949748235.1	0.1	98.7	98.4	93.53	15.2	chromosome
Polydrusus cervinus	GCA_935413205.1	0.7	99.6	96.8	92.66	72.8	chromosome
Polydrusus tereticollis	GCA_963920685.1	1.4	-	92.1	88.66	-	-
Rhynchophorus ferrugineus	GCA_014462685.1	0.6	99.1	96.7	93.06	0.5	scaffold
Sitophilus oryzae	GCF_002938485.1	0.8	99.0	97.3	92.83	2.8	scaffold
Taphrorychus bicolor	GCA_951812265.1	0.6	99.5	95.4	92.51	48.2	chromosome
Apoderus coryli	GCA_911728435.2	0.4	99.8	98.9	93.76	23.6	chromosome
Cylas formicarius	GCA_029955315.1	0.4	99.2	80.0	-	-	-
Chrysoperla carnea	GCA_905475395.1	0.6	97.9	98.1	94.65	94.4	chromosome

Table S2: Functions of genes convergently expanded or contracted in different species (Overlap). Methods: CAFE5, cafeplotter, R, eggnog. Table with list of orthogroups convergently expanded or contracted in the indicated species. Each item of the list is the most common assigned function of one orthogroup. Number of gene functions does not correspond to number of genes in overlaps, as not all genes were annotated by eggnog. Orthogroups present in more than one overlap are shown in each overlap. Refer to Table S1 for full species names.

**Overlap**	**Gene function**
Contracted *P. cylindrus*, *H. hampei*, Scolytinae anc	- Lipase / Phosphatidylcholine 1-acylhydrolase activity
Contracted *P. cylindrus*, *H. hampei*	- Zinc finger, C2H2 type<br>- Endonuclease/Exonuclease/Phosphatase family / Ribonuclease H protein<br>- Protein heterodimerization activity / Histone 2A<br>- AAA domain<br>- Choline dehydrogenase activity / GMC oxidoreductase<br>- Lipase / Phosphatidylcholine 1-acylhydrolase activity<br>- Serine-type endopeptidase activity<br>- Peptidase S1 family<br>- Catalyzes the reduction of fatty acyl-CoA to fatty alcohols<br>- Acyl-Coenzyme A oxidase<br>- Protein tyrosine phosphatase activity<br>- Ligase activity<br>- Skeletal muscle satellite cell migration / Craniofacial development protein<br>- Craniofacial development protein<br>- Transmembrane transporter activity / Sugar (and other) transporter<br>- Cellular response to interferon-beta Glutathione S-transferase<br>- Group 7 allergen<br>- Rho guanyl-nucleotide exchange factor activity<br>- MreB/Mbl protein / Heat shock 70 kDa protein
Contracted Scolytinae anc, *H. hampei*	- Lipase / Phosphatidylcholine 1-acylhydrolase activity<br>- Peptidase S1 family
Contracted Scolytinae anc, *P. cylindrus*	- Lipase / Phosphatidylcholine 1-acylhydrolase activity<br>- DDE superfamily endonuclease<br>- THAP<br>- DNA binding<br>- Amiloride-sensitive sodium channel (TC 1.A.6) family<br>- Odorant receptor
Expanded *P. cylindrus*,	- Heme binding / Cytochrome P450

H. hampei	- DNA replication factor
Expanded Scolytinae anc, *H. hampei*	- Spectrin repeats<br>- Immunoglobulin domain<br>- Converts alpha-aldose to the beta-anomer / Aldose 1-epimerase
Expanded *P. cylindrus* contracted *H. hampei*	- Nucleosomal DNA binding / Histone H3<br>- Core component of nucleosome<br>- K02A2.6-like<br>- Protein of unknown function (DUF3421)

Table S3: Functions of the top 10 genes under relaxed and under intensified selection in the Scolytinae ancestor, *Hypothenemus hampei* and *Platypus cylindrus*. Broad function contains a manual grouping of proteins with similar functions, Protein/Function contains a list of shortened output of eggnog functional annotation where each item of the list corresponds to an orthogroup. $log_2(k)$ shows the intensification and relaxation parameter from the RELAX analysis, positive values indicate intensification of selection, negative values indicate relaxation of selection.

Broad function	Protein/Function	$log_2(k)$
Top 10 genes under relaxed selection		
Gene expression regulation	- EF-1 guanine nucleotide exchange domain / Translation elongation factor activity<br>- Regulation of transcription, DNA-templated / T-box<br>- HELICc2 / Nucleobase-containing compound metabolic process<br>- rRNA adenine N(6)-methyltransferase family / Ribosomal RNA adenine dimethylases	-3.138224<br>-1.756162<br>-1.553601<br>-1.494108
Neural plasticity	- Dystrobrevin binding protein 1<br>- KIF-1 binding protein C terminal / Protein KBP homolog	-1.799216<br>-1.502370
Other	- C-terminal to LisH motif<br>- Unknown<br>- Protein binding, bridging<br>- Signal transducer activity	-2.714161<br>-1.774458<br>-1.578051<br>-1.505428
Top 10 genes under intensified selection		
Gene expression regulation	- LSM4 homolog, U6 small nuclear RNA / snRNP Sm proteins<br>- Found in Pit-Oct-Unc transcription factors / Regulation of transcription<br>- Regulation of mRNA processing	4.244409<br>1.345543<br>1.024210

Cytoskeleton/chromatin conformation	- Belongs to the actin family	1.187332
Protein transport	- ADP-ribosylation factor family / Protein transport<br>- Peptidase S24-like / Signal peptide processing	1.636761<br>0.939215
Electron transfer	- ATP-dependent (S)-NAD(P)H-hydrate dehydratase<br>- Ubiquinol-cytochrome C chaperone	0.815977<br>0.814161
Carbohydrate metabolism	- Chitin binding / Chitin metabolic process<br>- N-acetylgalactosaminyltransferase	0.676511<br>0.661514

Table S4: Functions of genes under positive selection in the Scolytinae ancestor, *Hypothenemus hampei* and *Platypus cylindrus*. List of functions per single copy orthogroup, where each item of the list corresponds to one orthogroup. *Ankyrin repeat domain-containing protein was only found to be under positive selection once, but could be grouped with different functional groups.

H. hampei	
Microtubule related processes	- Moesin ezrin radixin homolog
P. cylindrus	
Transcription regulation	- Sequence-specific DNA binding. It is involved in the biological process described with regulation of transcription, DNA-templated<br>- Zinc finger protein 512B<br>- HMG (high mobility group) box
Other	- It is involved in the biological process described with spermatogenesis
**Scolytinae ancestor**	
Gene expression regulation	- Nucleic acid binding. It is involved in the biological process described with regulation of transcription, DNA-templated<br>- Nucleic acid binding / RNA recognition motif<br>- Integrator complex subunit<br>- tRNA wobble uridine modification<br>- Mitochondrial ribosomal protein, L48<br>- Ankyrin repeat domain-containing protein *
DNA related processes	- ATP-dependent helicase activity<br>- IMP cyclohydrolase activity. It is involved in the biological process described with purine nucleotide biosynthetic process<br>- DNA helicase activity. It is involved in the biological process described with DNA replication initiation

Protein related processes	- Ankyrin repeat and FYVE domain-containing protein<br>- It is involved in the biological process described with proteolysis<br>- Cysteine-type endopeptidase activity. It is involved in the biological process described with proteolysis<br>- Binds to the signal sequence of presecretory protein when they emerge from the ribosomes and transfers them to TRAM (translocating chain-associating membrane protein)<br>- Unfolded protein binding. It is involved in the biological process described with protein folding
Calcium homeostasis	- Calreticulin family<br>- Spermidine synthase tetramerisation domain<br>- Ankyrin repeat domain-containing protein *
Microtubule related processes	- ATP-dependent microtubule severing protein. Stimulates microtubule minus-end depolymerization and poleward microtubule flux in the mitotic spindle. Regulates microtubule stability in the neuromuscular junction synapse. Involved in lipid metabolism by regulating the size and distribution of lipid droplets. Involved in axon regeneration by regulating microtubule severing<br>- WASH complex subunit 7, N-terminal<br>- Belongs to the TRAFAC class myosin-kinesin ATPase superfamily. Myosin family<br>- Ankyrin repeat domain-containing protein *<br>- Kinase activity. It is involved in the biological process described with DGKQ
Respiratory electron chain	- Component of the ubiquinol-cytochrome c reductase complex (complex III or cytochrome b-c1 complex), which is a respiratory chain that generates an electrochemical potential coupled to ATP synthesis<br>- This is a component of the ubiquinol-cytochrome c reductase complex (complex III or cytochrome b-c1 complex), which is part of the mitochondrial respiratory chain. This protein may mediate formation of the complex between cytochromes c and c1
Ion binding	- Zinc ion binding<br>- Metal ion binding<br>- Metal ion binding

	- Heme binding. It is involved in the biological process described with response to oxidative stress
Other	- Transferase activity, transferring glycosyl groups<br>- DDRGK<br>- GTP binding<br>- Essential subunit of the N-oligosaccharyl transferase (OST) complex which catalyzes the transfer of a high mannose oligosaccharide from a lipid-linked oligosaccharide donor to an asparagine residue within an Asn-X-Ser Thr consensus motif in nascent polypeptide chains

Statistical analysis

Table S5: Model comparison of PGLS models with Brownian motion model or OU model. Models tested: $Brownian : median\,k \sim parental\,care, correlation = corBrownian(1, tree, form =\sim species)$. $OU : median\,k \sim parental\,care, correlation = corMartins(1, tree, form =\sim species)$. df = degrees of freedom, AIC = Akaike information criterion, BIC = Bayesian information criterion, logLik = log Likelihood, L.Ratio = likelihood ratio.

	Model	df	AIC	BIC	logLik	Test	L.Ratio	p-value
Brownian	1	5	-18.94348	-15.40323	14.47174			
OU	2	6	-29.03419	-24.78589	20.51710	1 vs 2	12.09071	5e-04

Table S6: Estimated marginal means for each parental care type in the PGLS with OU model. emmean = estimated marginal mean, SE = standard error, df = degrees of freedom, CL = confidence interval. Degrees-of-freedom method: satterthwaite, confidence level used: 0.95.

Parental care	emmean	SE	df	lower.CL	upper.CL
egg attendance	1.008	0.0424	13.0	0.917	1.100
nest building	1.102	0.0373	13.9	1.021	1.182
no care	1.018	0.0334	13.9	0.946	1.089
larval attendance	0.875	0.0528	13.9	0.762	0.988

Table S7: Contrasts between parental care types in the PGLS with OU model. estimate = estimated difference in means, SE = standard error, df = degrees of freedom, CL = confidence interval, t.ratio = t statistic. Degrees-of-freedom method: satterthwaite, P value adjustment: fdr method for 6 tests.

Contrast	estimate	SE	df	t.ratio	p-value
egg attendance - nest building	-0.09328	0.0565	13.4	-1.651	0.1464
egg attendance - no care	-0.00939	0.0540	13.4	-0.174	0.8645
egg attendance - larval attendance	0.13316	0.0677	13.6	1.966	0.1402
nest building - no care	0.08389	0.0501	13.9	1.675	0.1464
nest building - larval attendance	0.22644	0.0647	13.9	3.501	0.0213
no care - larval attendance	0.14255	0.0625	13.9	2.281	0.1163

Figures

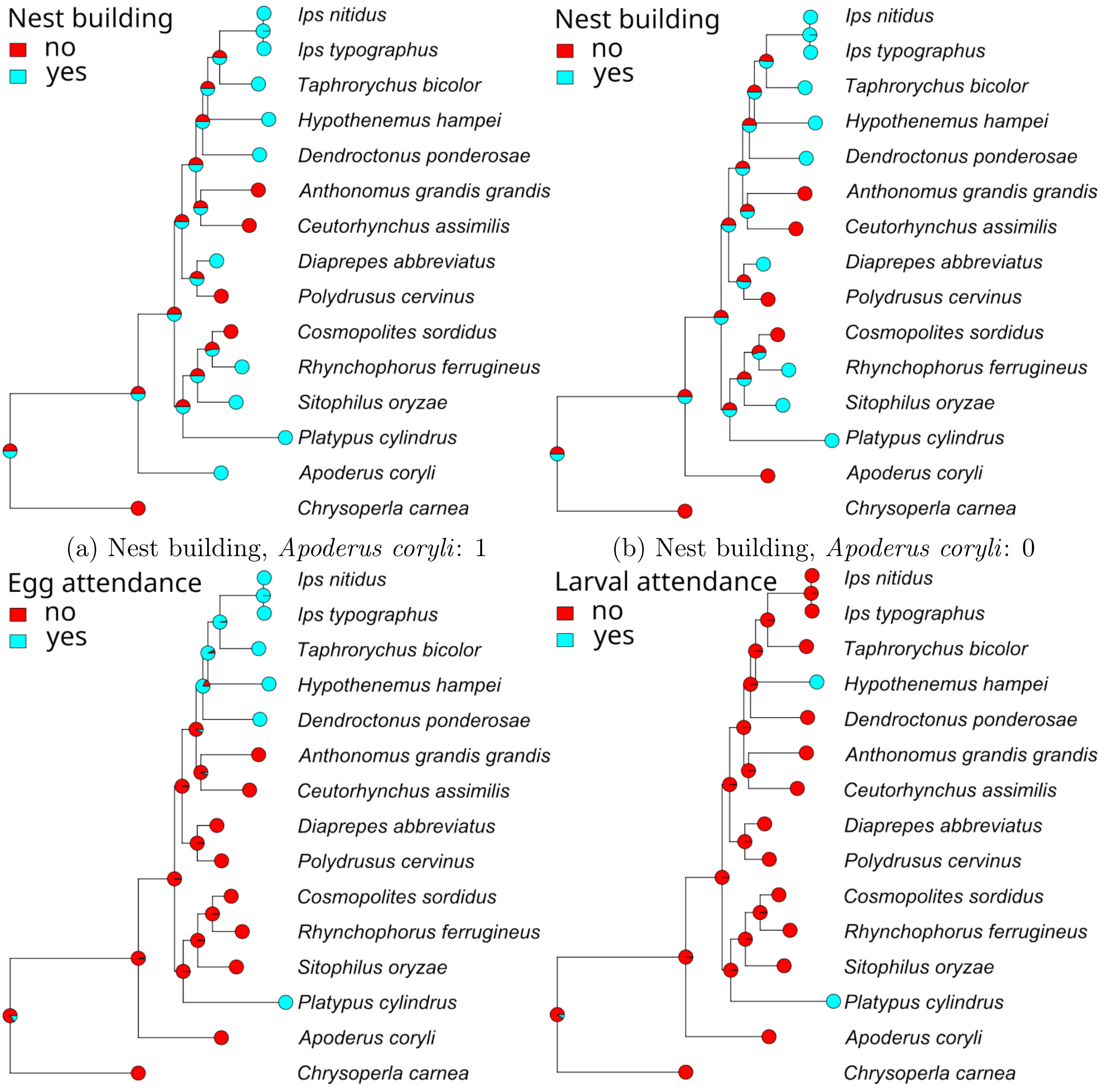

(a) Nest building, *Apoderus coryli*: 1

(b) Nest building, *Apoderus coryli*: 0

(c) Egg attendance

(d) Larval attendance

Figure S1: Ancestral reconstruction of weevil care traits. The closest outgroup of Curculionidae contains species with and without nest building. To test whether the state in this outgroup has an impact on the ancestral reconstruction of nest building within Cuculionidae, both states (0 - no nest building, 1 - nest building) were plotted.

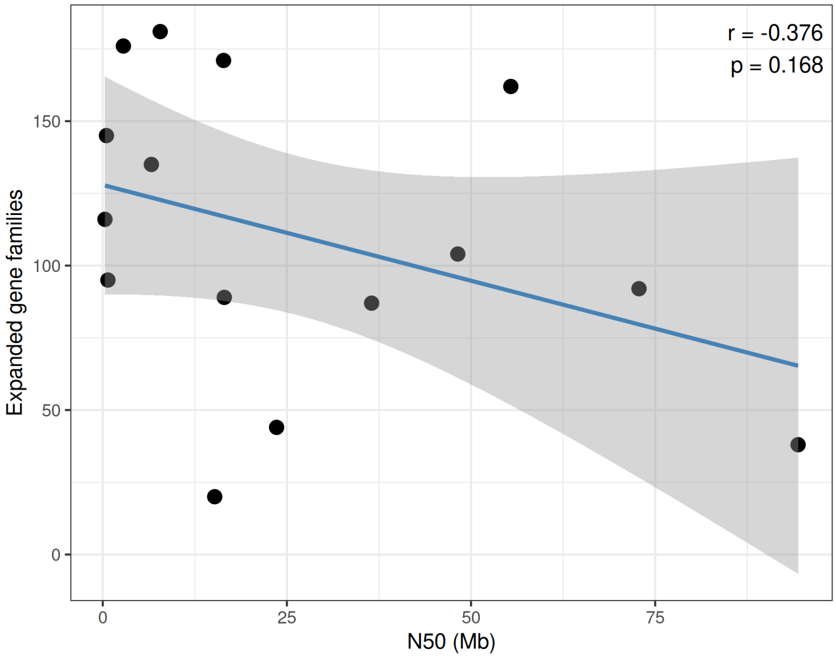

(a) Expansions

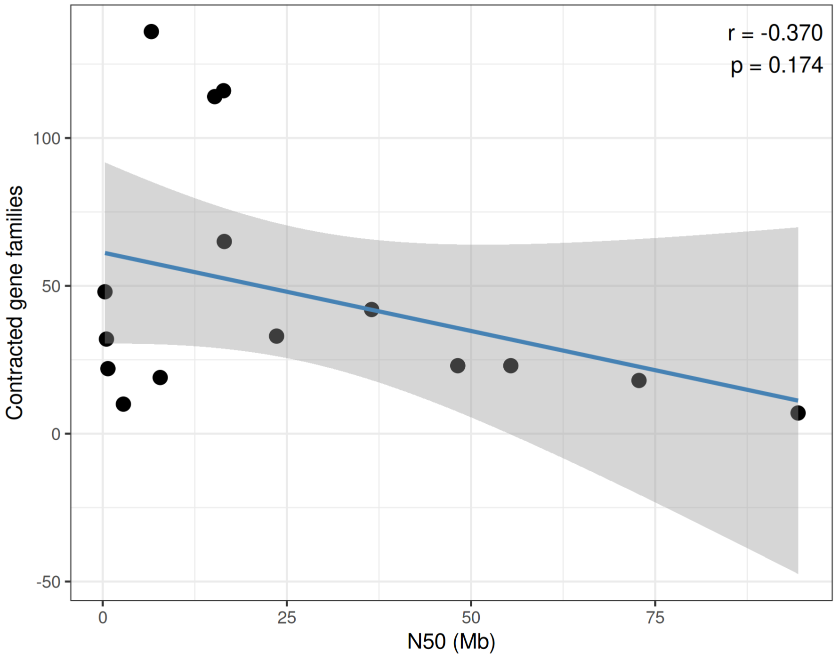

(b) Contractions

Figure S2: Relationship between contiguity metric N50 and significantly expanded or contracted gene families.

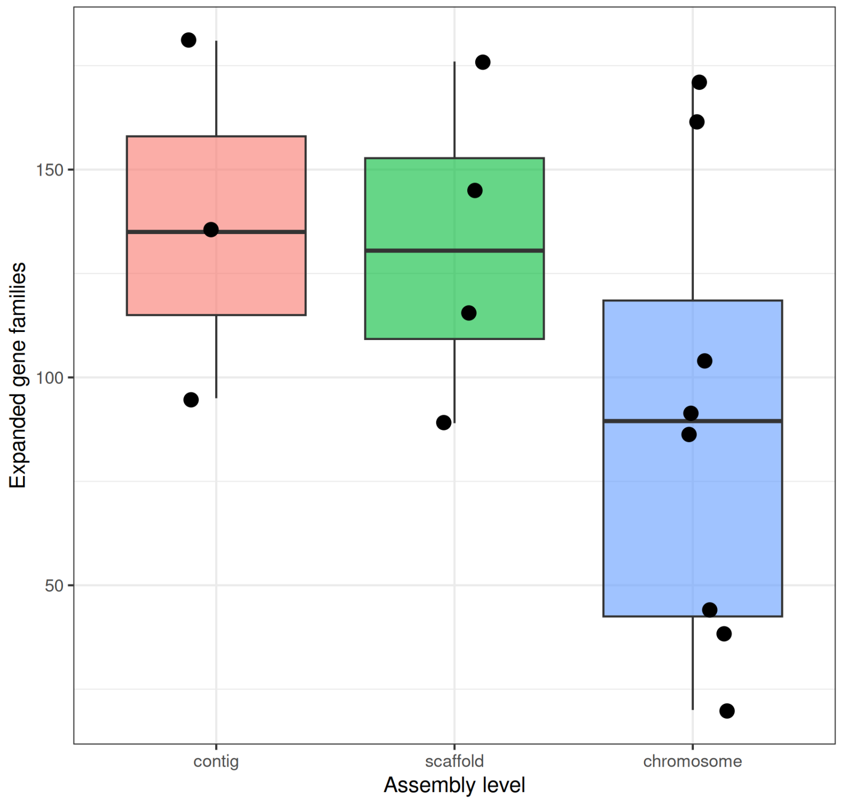

(a) Expansions

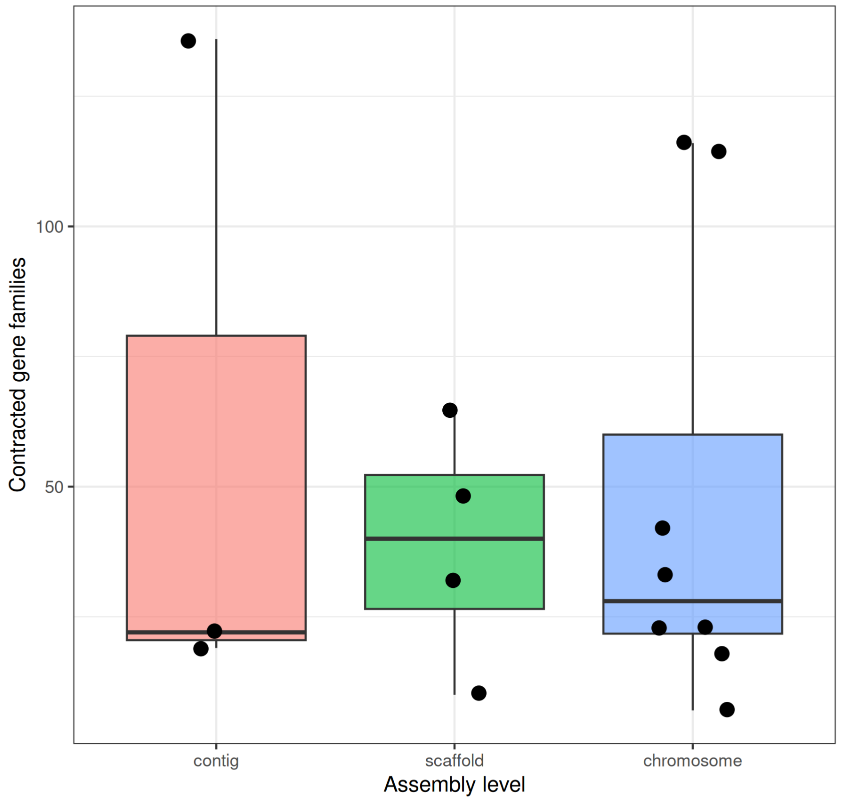

(b) Contractions

Figure S3: Relationship between assembly level (contig, scaffold, or chromosome) and significantly expanded (degrees of freedom = 2, F-statistic = 1.483, p-value = 0.266) or contracted (degrees of freedom = 2, F-statistic = 0.179, p-value = 0.838) gene families. Numbers above brackets show p-values for the indicated comparison.

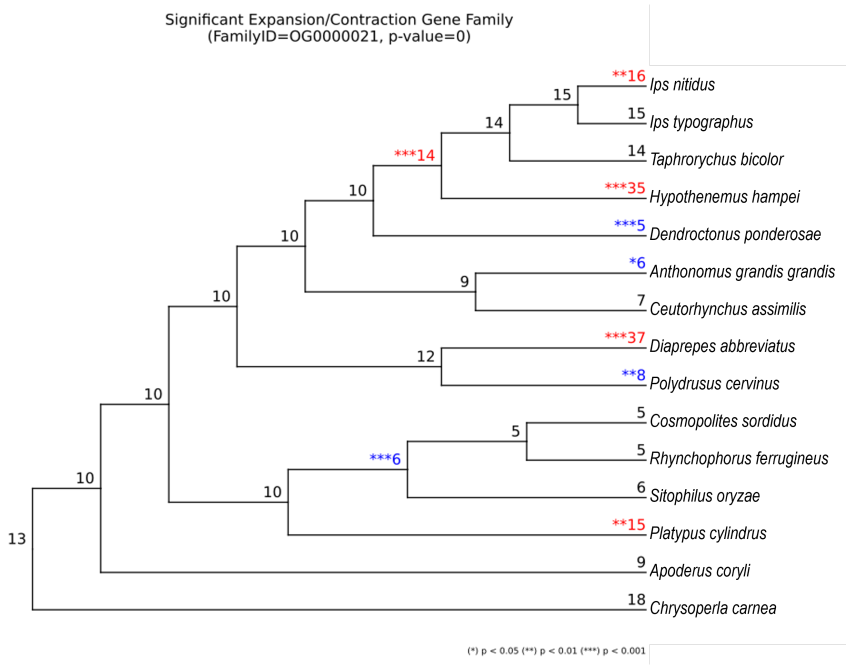

Figure S4: Gene family size evolution of the orthogroup annotated as Heme binding / Cytochrome P450. Red numbers indicate significant expansions, blue numbers indicate significant contractions. This gene family is significantly expanded in both, Hypothenemus hampei (35 gene copies) and Platypus cylindrus (15 gene copies). However, it is also significantly expanded in two other species and one ancestral branch.

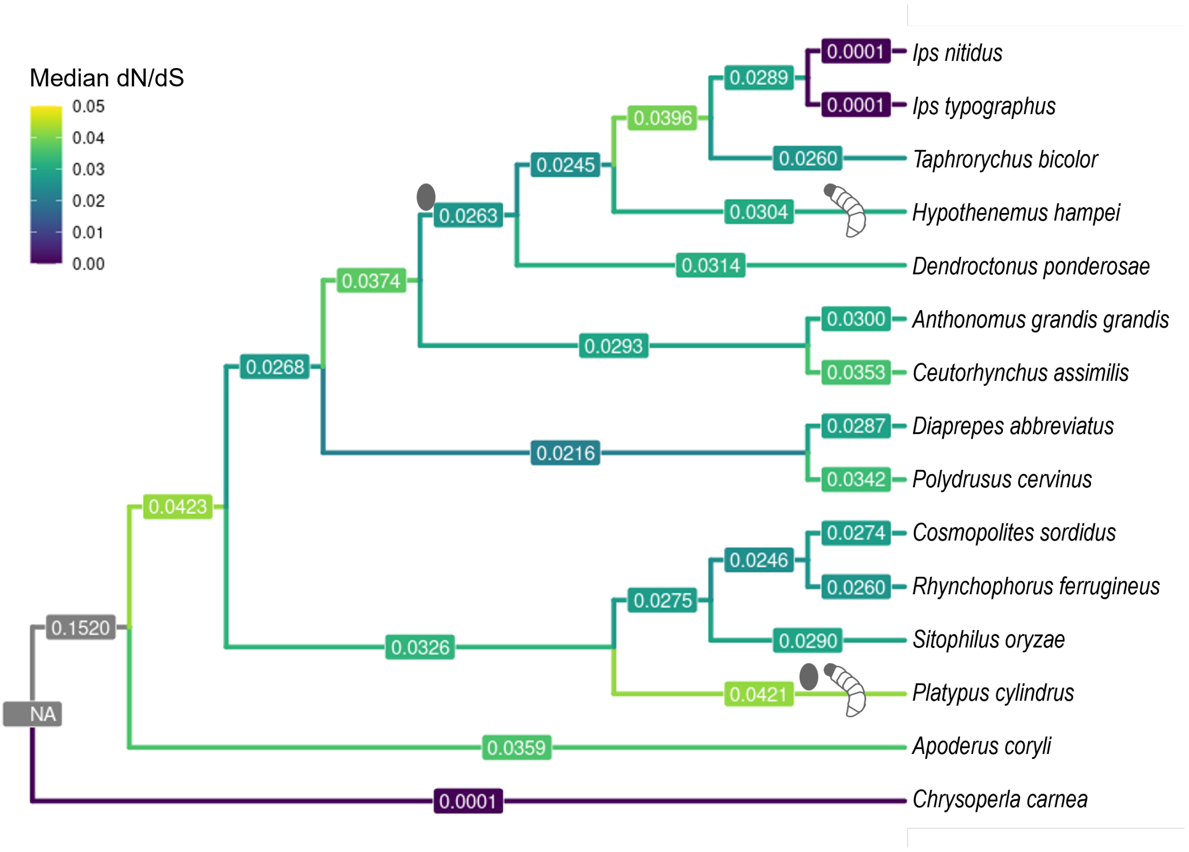

Figure S5: Median dN/dS tree. dN/dS was calculated for each single copy orthologue using CodeML, the median per branch was then calculated and plotted. A dN/dS value below 1 indicates purifying selection, above 1 positive selection. Altogether, selection is strongly purifying across the tree without large differences between the branches.

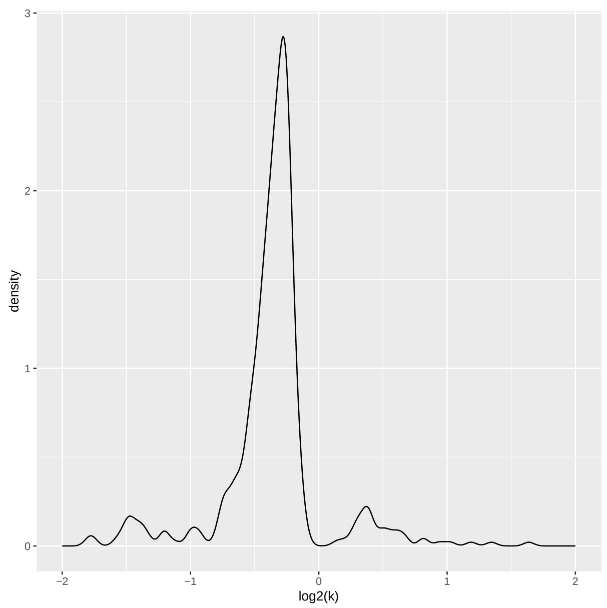

Figure S6: Relax: distribution of log2(k): Below zero - relaxation, above zero - intensification. Here, the change of intensity of selection in the three focal branches (*Hypothenemus hampei*, *Platypus cylindrus*, Scolytinae ancestor) compared to all background branches is analysed

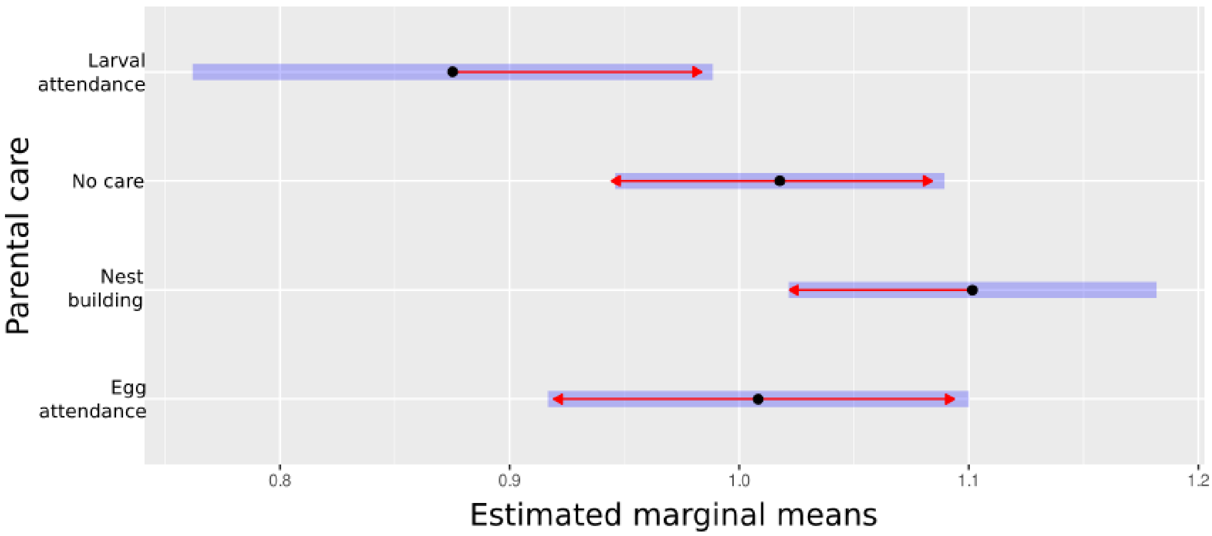

Figure S7: Contrasts of estimated marginal means of median k from the PGLS with OU model.

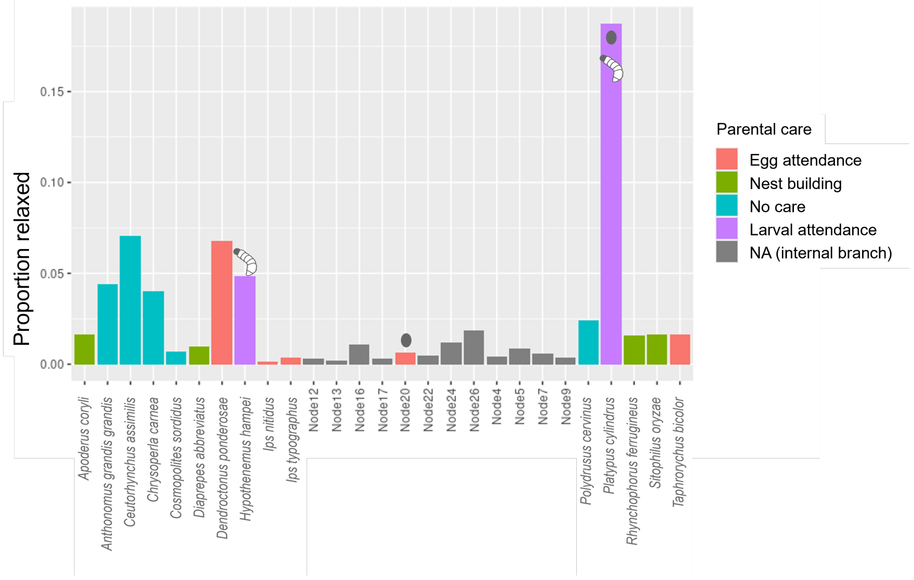

(a) Relaxed

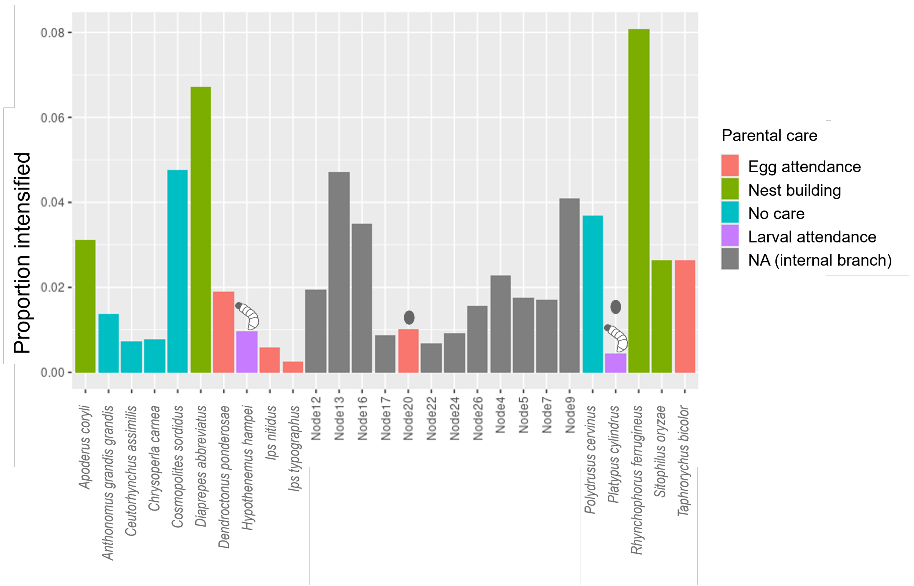

(b) Intensified

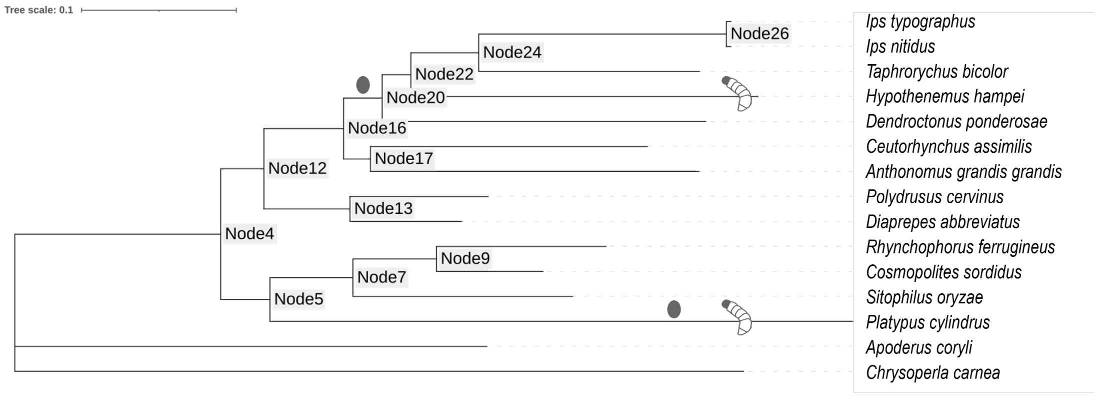

(c) Node labels

Figure S8: Proportion of orthogroups under relaxed selection on each branch. log2(k) was calculated for all single copy orthologues for each branch separately. For each branch, the median value of all significant (p.adjust) k values was then calculated.
